# Cost-effectiveness analysis of anlotinib versus sunitinib as first-line treatment for metastatic renal cell carcinoma in China

**DOI:** 10.1371/journal.pone.0281402

**Published:** 2023-02-07

**Authors:** Jingyang Lin, Qingxia Fang, Xiaochun Zheng

**Affiliations:** 1 Department of Cardiovascular Medicine, Heart Center, Zhejiang Provincial People’s Hospital (Affiliated People’s Hospital, Hangzhou Medical College), Hangzhou, Zhejiang, China; 2 Department of Pharmacy, Center for Clinical Pharmacy, Cancer Center, Zhejiang Provincial People’s Hospital (Affiliated People’s Hospital, Hangzhou Medical College), Hangzhou, Zhejiang, China; The University of Texas MD Anderson Cancer Center, UNITED STATES

## Abstract

**Background:**

Sunitinib was approved several years ago as a first-line drug for treating metastatic renal cell carcinoma (mRCC); however, its high price and broad side effects when administered at the standard dose have limited its clinical use. A clinical trial (NCT02072031) confirmed that anlotinib could be used as the first-line treatment for mRCC. This study was conducted to evaluate the cost-effectiveness of anlotinib as a first-line treatment for mRCC compared to that of sunitinib in China.

**Methods:**

A Markov model was established to compare the cost-effectiveness of anlotinib with that of sunitinib. Clinical data were obtained from a multi-center phase II trial (clinical trial information: NCT02072031). Utility values were obtained from the literature. Total costs were calculated from a Chinese societal perspective. A sensitivity analysis was conducted to assess the model uncertainty. The life-year (LY), quality-adjusted life-year (QALY), and incremental cost-effectiveness ratio were calculated.

**Results:**

The base-case analysis over a lifetime horizon of 10 years showed that the anlotinib group had 2.196 LYs and 1.487 QALYs at a total cost of $68,597.84. The sunitinib group had 2.194 LYs and 1.432 QALYs at a total cost of $88,060.02. This resulted in incremental cost-effectiveness ratios (ICER) of anlotinib versus sunitinib of $-9,210,858.93 per LYs and $-354,117.07 per QALYs, suggesting that anlotinib is a more effective and less costly strategy than sunitinib.

**Conclusion:**

Anlotinib may be a more cost-effective first-line treatment strategy for mRCC than sunitinib in China.

## Introduction

Renal cell carcinoma (RCC) accounts for more than 90% of all malignant renal tumors. In most cases, the clinical features of RCC are unclear. Because RCC is insensitive to chemotherapy and radiotherapy, surgical resection is the only treatment for this disease. Metastasis greatly reduces the survival rate of patients. The 5-year survival rates of patients with localized, regional, and metastatic RCC (mRCC) are 92.5%, 69.6%, and 12.0%, respectively [1]. Marked advances have been made in the treatment of mRCC in the past decade, including the development of numerous targeted drugs such as sunitinib, sorafenib, pazopanib, temsirolimus, and bevacizumab plus interferon-α [2–6]. In recent years, interferon-α has been replaced by the tyrosine kinase inhibitors sunitinib and pazopanib as first-line standard treatments for mRCC in China [7]. However, these treatments are costly, making them unaffordable for low-income patients.

Anlotinib (AL3818) is a new oral tyrosine kinase inhibitor that targets VEGFR, FGFR, PDGFR, RET, and c-Kit to inhibit angiogenesis and tumor proliferation. This drug was approved for marketing in May 2018 in China [8]. A multi-center phase II clinical trial (clinical trial information: NCT02072031) was performed to evaluate the efficacy and safety of anlotinib as a first-line treatment for mRCC [9]. A total of 133 volunteers were enrolled in the study, with 90 patients in the anlotinib group and 43 patients in the sunitinib group. Both groups showed a similar median overall survival (OS) (30.9 vs. 30.5 months, P > 0.05), progression-free survival (PFS) (17.5 vs. 16.6 months, P > 0.05), objective response rate (30.3% vs. 27.9%), and 6-week disease control rate (97.8% vs. 93.0%, P = 0.33). These results suggested that anlotinib is a favorable choice for patients with mRCC. Based on this research, the guideline of Chinese Society of Clinical Oncology (CSCO) for RCC recommended anlotinib as a first-line treatment for patients with mRCC.

However, the high price of this new treatment is an important factor that must be considered in cancer therapy. The increasing costs of cancer treatment have highlighted the need for a cost-effectiveness analysis to enable policymakers to make more efficient use of limited resources. Therefore, it is interesting to evaluate whether anlotinib is cost-effective as a first-line treatment for mRCC in China. For all we know, this study is the first cost-effectiveness comparison between anlotinib and sunitinib for first-line treatment of mRCC.

## Materials and methods

### Patients and treatments

Clinical data were obtained from a multi-center phase II randomized controlled clinical trial performed to compare first-line treatment with anlotinib and sunitinib in patients with mRCC (clinical trial information: NCT02072031) [9]. Anlotinib was administered orally once daily at a dose of 12 mg (continuous administration for two weeks and withdrawal for one week). Sunitinib was administered orally once daily at a dose of 50 mg (continuous administration for four weeks and withdrawal for two weeks). Treatment was discontinued when the disease progressed, toxic effects were intolerable, or informed consent was withdrawn during the double-blind phase. The clinical outcomes of PFS, OS, and adverse events (AEs) (grade ≥3) are shown in Table 1.

**Table 1:** Clinical outcomes and most common AEs (≥3 grade).

Variables	Values	
	Anlotinib	Sunitinib
**Clinical efficacy (months)**		
**Median OS**	30.9	30.5
**Median PFS**	17.5	16.6
**Total AEs (grade ≥3) (%)**	28.9	55.8
**Adverse event**		
**Hypertension**	13.3	25.6
**Hand-foot syndrome**	3.3	14
**Diarrhea**	1.1	0
**Stomatitis**	2.2	2.3
**Laboratory abnormality**		
**Increased triglyceride**	4.4	7.0
**Increased cholesterol**	1.1	2.3
**Increased creatinine**	0	2.3
**Hypophosphatemia**	3.3	9.3
**Thrombocytopenia**	0	11.6
**Increased alkaline phosphatase**	2.2	0
**Leukopenia**	0	2.3
**Anemia**	2.2	4.7
**Neutropenia**	0	9.3
**Lymphopenia**	0	4.7

OS, overall survival; PFS, progression-free survival; AE, adverse event

### Model structure

**Fig 1:**
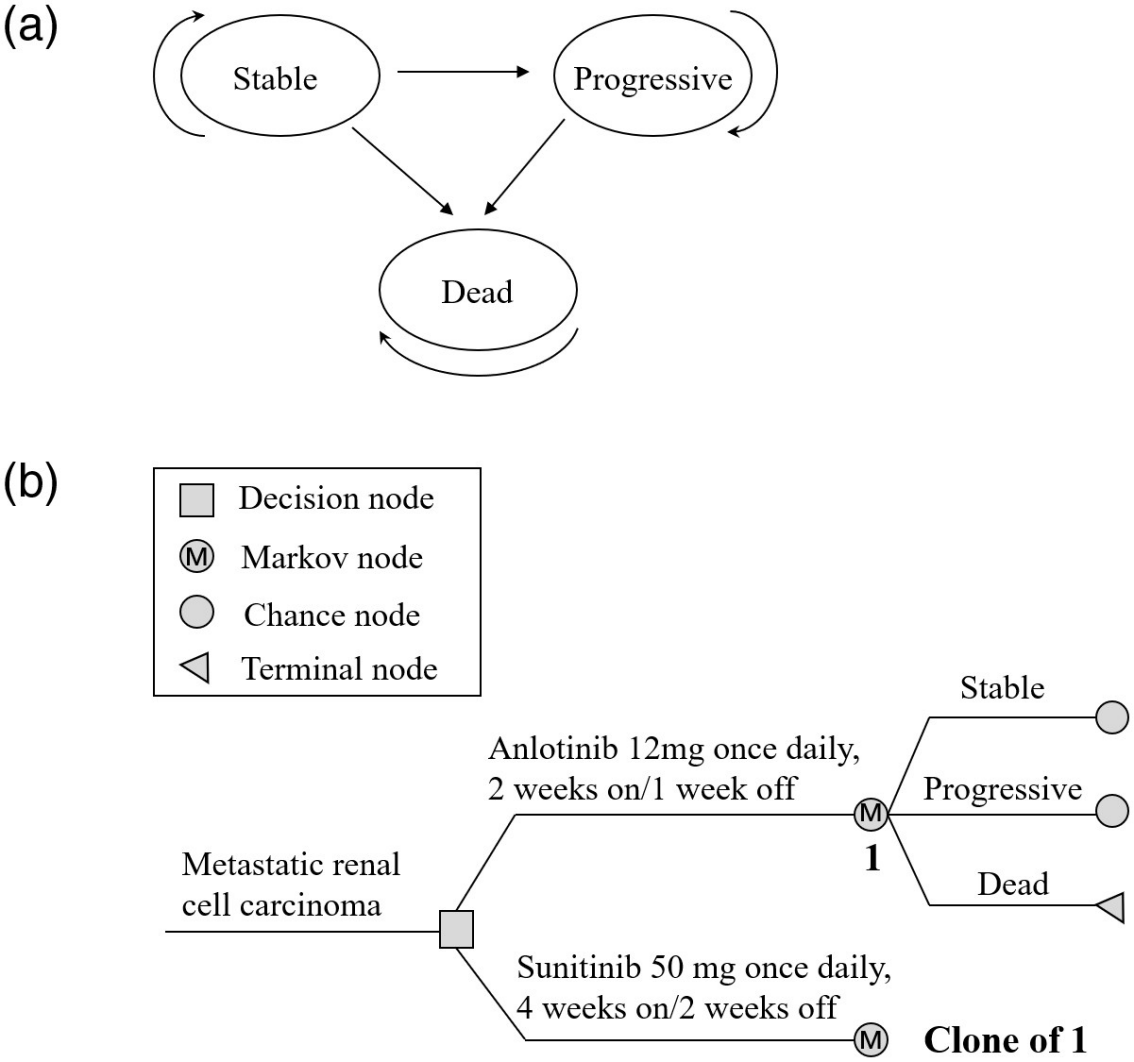
A markov model for mRCC. (a) Three transitional health states. (b) Markov model used to compare two treatment strategies for mRCC.

A Markov model was developed using TreeAge Pro 2011 software (TreeAge Software, Williamstown, MA, USA) to evaluate the cost-effectiveness of anlotinib. As shown in Fig 1, three health states were included in our model: stable, progressive, and dead. Progression from the stable state, death from the stable state, and mortality after progression were estimated based on survival functions for PFS, OS, and progressive disease (PD, defined as the interval between the date of PFS and the date of OS). We hypothesized that all patients started in a stable state and were treated with anlotinib or sunitinib until disease progression or unacceptable AEs occurred. Patients in a progressive state can receive subsequent treatment until death. Patients in both the progressive and stable states were at risk of dying and could only transition from one state to another state or remain in the same state for a cycle. The transition probabilities for each cycle were calculated as follows: P_(1 month)_ = 1−(0.5)^(1/median time to event)^, P = 1-e^-R^, and R = -ln(0.5)/(time to event/number of treatment cycles) (Table 2) [10, 11]. According to the China Guidelines for Pharmacoeconomic Evaluations, health utilities and costs are discounted at a rate of 5% annually [12]. The model covered a cycle of one month and time horizon of 10 years, considering that all patients were expected to have died within 10 years. This is consistent with data from the pivotal trial of sunitinib, in which patients randomized to sunitinib showed a median OS of 26.4 months (95% confidence interval: 23.0–32.9 months), and that from the American Cancer Society revealing a 5-year survival rate of 8% for patients with stage IV cancer [13]. The primary model outputs included the total costs, life years (LYs), quality-adjusted life years (QALYs), and incremental cost-effectiveness ratio (ICER).

**Table 2:** Key parameters input in the model.

Variable	Value	Range	Distribution	Reference
**Transition probabilities**				
**Anlotinib group**				
**Progression from stable state**	0.039	0.031 to 0.047	Beta	[9]
**Death from stable state**	0.022	0.018 to 0.026	Beta	[9]
**Mortality after progression**	0.050	0.040 to 0.061	Beta	[9]
**Sunitinib group**				
**Progression from stable state**	0.041	0.033 to 0.049	Beta	[9]
**Death from stable state**	0.022	0.018 to 0.026	Beta	[9]
**Mortality after progression**	0.049	0.039 to 0.059	Beta	[9]
**Utilities**				
**Stable state**	0.730	0.584 to 0.876	Beta	[15–17]
**Progressive state**	0.660	0.528 to 0.792	Beta	[15–17]
**Disutility due to AEs (grade ≥3)**	0.157	0.126 to 0.188	Beta	[15–17]
**Costs per cycle, $**				
**Anlotinib**	864.76	691.81 to 1037.71	Gamma	Local charge
**Sunitinib**	1830.48	1464.38 to 2196.58	Gamma	Local charge
**Societal costs**	54.58	43.66 to 65.50	Gamma	[14]
**Tests**	248.96	199.17 to 298.75	Gamma	Local charge
**Outpatient fees**	6.32	5.06 to 7.58	Gamma	Local charge
**Grade ≥3AEs in anlotinib group**	23.76	19.01 to 28.51	Gamma	Expert Opinion
**Grade ≥3AEs in sunitinib group**	265.44	212.35 to 318.53	Gamma	Expert Opinion
**Costs for progressive state**	4535.08	3628.06 to 5442.10	Gamma	[15]

### Costs and adverse events inputs

Costs included both direct medical and societal costs, estimated from a societal perspective in China. Direct medical costs included the following: drugs, laboratory tests, treatment of AEs (grade ≥3), and outpatient fees. The prices of drugs and laboratory tests were obtained from Zhejiang Provincial People’s Hospital. The occurrence rate of AEs was taken from the clinical trial. Management strategies for grade ≥3 AEs were based on expert opinions and clinical practice. The costs of treating AEs were calculated based on patient records from the local hospital. In addition, societal costs, including travel and time (loss of wages), were calculated according to China’s median monthly salary [14]. Anlotinib and sunitinib were predicted to have the same average societal costs. All costs were adjusted to USD ($1 = RMB 6.3017, CNY Central Historical Parity Rate 2022) (Table 2).

### Utilities

Health utility ranged from 1 (complete health) to 0 (death). This index converts one year of life lived with the disease into a QALY. One QALY represents the number of years of a completely healthy life. The mean health utility values for the stable and progressive states obtained from published literature were 0.730 and 0.660, respectively, assuming that the two groups had the same utility values after progression. The model included disutility values due to grade ≥3 AEs [15–17].

### Cost-effectiveness

A cost-effectiveness analysis was performed using the ICER. According to the World Health Organization guidelines for cost-effectiveness analysis, the effects of variables on the incremental net health benefit were examined using 3× China’s gross domestic product (GDP) per capita in 2021 ($34,340.50) as the willingness-to-pay (WTP) threshold [18]. Anlotinib was regarded as an economical selection compared to sunitinib if the ICER ≤ $34340.50; for costs above this value, anlotinib was not regarded as an economical option [19].

### Sensitivity analysis

The key model parameters (low/high values) were verified using one-way sensitivity analysis (Table 2). Except for discount rates ranging from 0 to 8%, univariate sensitivity analysis was performed for ±20% of one variable, whereas the other variables remained unchanged. The results are presented in tornado diagrams.

The uncertainty of all parameters was evaluated using probabilistic sensitivity analysis (PSA), and the results are presented in a cost-effectiveness acceptability curve (CEAC). PSA was performed using 1000 Monte Carlo simulations, with all parameters varying simultaneously with a specific distribution pattern. Different distributions were assigned to model parameters (beta distributions for probabilities and utilities and gamma distributions for costs) [20].

## Results

### Base case outcomes

Approximately 99% of patients in both groups died when the model simulation was terminated. Base-case analysis over a lifetime horizon of 10 years showed that the anlotinib group had 2.196 LYs and 1.487 QALYs at a total cost of $68,597.84. Sunitinib group had 2.194 LYs and 1.432 QALYs at a total cost of $88,060.02. The ICER of anlotinib vs. sunitinib was negative ($-9,210,858.93 per LY, $-354,117.07 per QALY). Because anlotinib was estimated to provide more QALYs at a lower cost than sunitinib, it was considered dominant to sunitinib in the base-case analysis. According to the recommendations of World Health Organization recommendations (WHO), it was notably economical when the ICER was below 1 ×the GDP per capita, not to mention the ICER was negative in our study. Therefore, anlotinib showed an absolute advantage over sunitinib as a first-line treatment for mRCC.

### Probabilistic sensitivity analyses results

**Fig 2:**
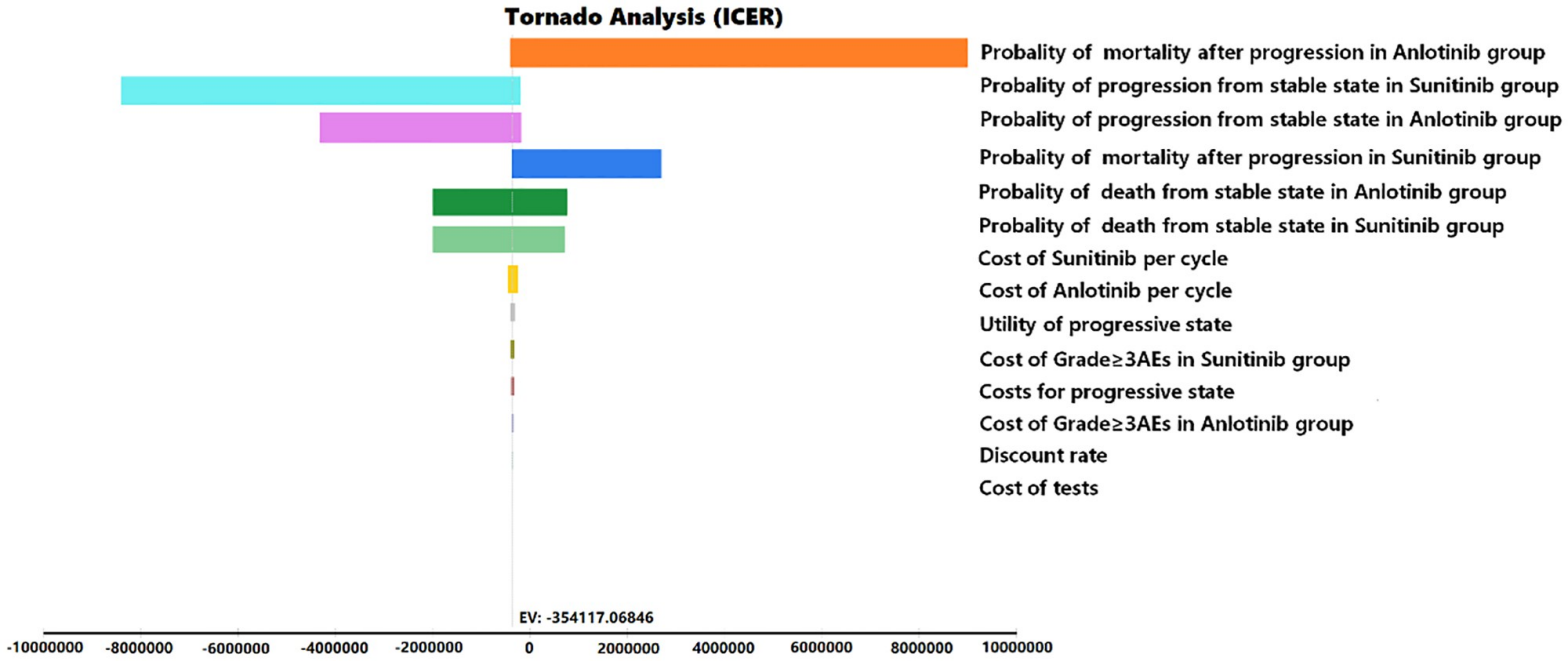
Tornado diagram shows incremental cost-effectiveness ratio (ICER) of anlotinib vs. sunitinib for different input parameters.

The tornado diagram drawn based on the results of one-way sensitivity analysis suggested that some parameters significantly impacted the net health benefits of anlotinib compared to those of sunitinib (Fig 2). The probability of mortality after progression in the anlotinib group was the most sensitive parameter affecting the ICER results, with a value range of $-44,592.55 to $596,431.60. The other major parameter in the model was the probability of progression from a stable state in the sunitinib and anlotinib groups. Other factors, such as the discount rate and testing costs, had little impact on the ICER.

**Fig 3:**
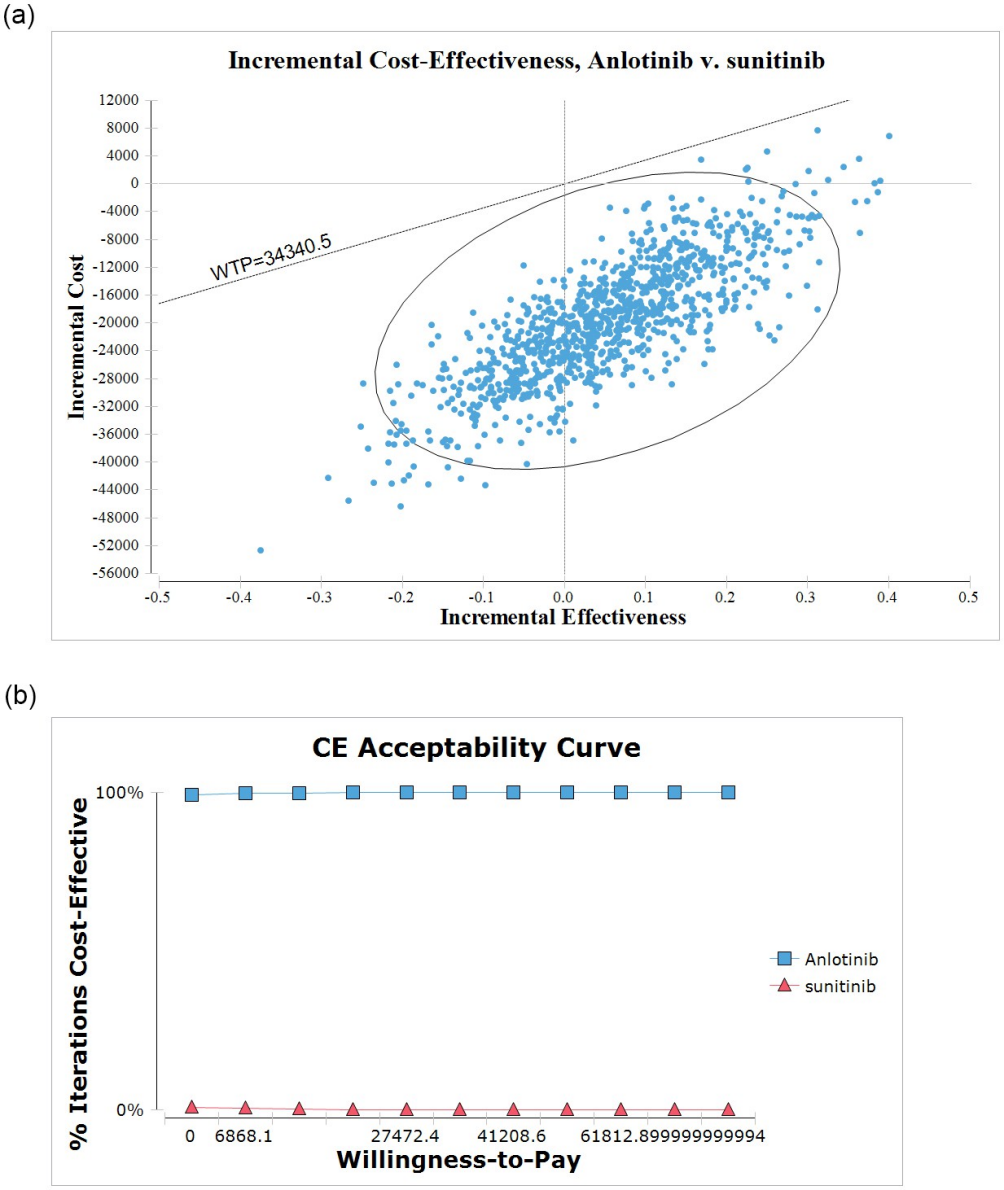
The results of probabilistic sensitivity analysis. (a) Scatterplot of the ICER between the anlotinib and sunitinib group. (b) The cost-effectiveness acceptability curves for anlotinb compared to the sunitinib at different willingness-to-pay thresholds.

PSA was conducted to examine the CEAC under different WTP levels. The probability of cost-effective treatment strategies typically does not change as the WTP increases. The CEAC and probabilistic scatter plots are shown in Fig 3. Although the WTP increased, anlotinib remained the preferred strategy. Regardless of the situation, the anlotinib group was more cost-effective in approximately 100% of simulations than the sunitinib group, with a WTP threshold of $34,340.5. Therefore, anlotinib showed an economic advantage over sunitinib as a first-line treatment for mRCC.

## Discussion

With the widespread clinical application of new targeted agents for mRCC, the survival rate and quality of life of patients have improved but have also led to a sharp increase in healthcare costs [21]. Because health resources are limited, it is necessary to evaluate the cost-effectiveness of first-line treatment recommendations for mRCC to balance the financial burden and health benefits. We performed a cost-effectiveness analysis of two first-line treatments for mRCC from a societal perspective in China. Anlotinib was estimated to provide more QALYs at a lower cost than sunitinib, and the ICER of anlotinib versus sunitinib was negative ($-354,117.07 per QALY). Our results suggest that anlotinib is a cost-effective first-line treatment strategy, as it is the dominant therapy according to both cost and QALYs. Anlotinib was also more beneficial than sunitinib at our WTP threshold. PSA also demonstrated a high likelihood of anlotinib being more cost-effective than sunitinib. These findings from a Chinese societal perspective may not apply to health jurisdictions outside of China.

We examined the cost-effectiveness of anlotinib versus that of sunitinib as a first-line treatment for mRCC based on PFS, OS, and AE incidence (grade ≥3) using data from a clinical trial, utility data from previous research reports, published cost estimates, and local charges. In the clinical trial, anlotinib showed similar efficacy but fewer side effects than sunitinib. However, the clinical trial did not report the 95% confidence intervals for PFS and OS; therefore, we applied a variation of ±20% of the parameters. The quality of life scores in the clinical trial did not differ between the two groups; thus, the utility values were the same in both groups.

An economic evaluation of anlotinib for treating malignant tumors has not been widely performed, and only two studies reported the results of cost-effectiveness analysis of anlotinib for small cell lung cancer (SCLC). Gong et al. evaluated the cost-effectiveness of anlotinib compared with that of a placebo as third- or further-line treatment for relapsed SCLC from a Chinese societal perspective [14]. In the study, anlotinib was estimated to result in an additional 0.12 QALYs at an incremental cost of $2131.32, resulting in an ICER of $17,741.94/QALY, which did not exceed the WTP in China. Fei et al. reported the cost-effectiveness results of anlotinib versus pembrolizumab and nivolumab as the third-line treatment in recurrent SCLC patients from the Chinese healthcare system perspective [22]. In the analysis by Fei et al., the likelihood of anlotinib being cost-effective was 87.5% to 99.9% at a willingness-to-pay (WTP) threshold of $11,144 to $33,431/QALY. These results demonstrated that anlotinib was a cost-effective treatment for patients with relapsed SCLC who experienced failure of at least two lines of chemotherapy in China. This finding is similar to the results of our study and indicates that anlotinib may improve the health outcomes of patients and allow for more efficient use of financial resources. However, at present, the indication of anlotinib for mRCC requires self-payment in China [23]. We suggest that it could be included in the medical insurance reimbursement policy to reduce the economic burden of patients.

However, our study had several limitations. First, the clinical data were primarily obtained from a relatively small clinical trial but not based on patient-level data in clinical practice. Second, the clinical results and costs in our study were obtained from the Chinese population, which may differ from those in other countries. Additionally, in actual clinical practice, many adjuvant treatments, such as Chinese herbal medicine and immunotherapy, can affect the total cost of cancer treatment. In China, Chinese herbal medicines are extensively used in clinical practice. Adjuvant treatments were excluded from this study to simplify the evaluation. In recent years, the monopoly on imported drugs in China’s pharmaceutical market has changed. Accordingly, the price of targeted drugs has greatly decreased, and the share of targeted drugs for mRCC in the Chinese market is growing. These data emphasize the importance of developing innovative drugs in China to ensure the coverage of affordable prescription drugs. Our results provide beneficial information for the rational use of drugs, selection of specific drugs for patients, and decision-making by government health policy-makers.

## Conclusions

We constructed a Markov model to explore the cost-effectiveness of anlotinib versus that of sunitinib in patients with previously untreated mRCC from a Chinese societal perspective. Anlotinib was the dominant therapy in terms of both cost and QALYs. Our findings support that anlotinib is a cost-effective first-line treatment option for mRCC in Chinese healthcare settings and may be recommended by the China National Health Service.

## Supporting information

S1 File (RAR)
